# The Hidden Epidemic: Post-Release Tuberculosis Risk in Formerly Incarcerated Populations

**DOI:** 10.21203/rs.3.rs-7707029/v1

**Published:** 2025-10-06

**Authors:** Megan Murray, Chuan-Chin Huang, Meredith Brooks, Mercedes Becerra, Roger Calderon, Carmen Contreras, Judith Jimenez, Leonid Lecca, Alicia Madden, Rosa Yataco, Zibiao Zhang

**Affiliations:** Harvard Medical School; Harvard School of Public Health; boston university; Harvard University; Universidad de San Martin de Porres; Socios En Salud; Socios En Salud; Socios En Salud; Harvard Medical School; Socios En Salud; Harvard School of Public Health

## Abstract

Incarcerated populations face an extremely high risk of tuberculosis (TB), yet little is known about whether this elevated risk persists after release into the community. Among 3,666 TB patients aged ≤ 60 years enrolled in a prospective cohort study in Lima, Peru, 188 (5%) reported a history of incarceration. These individuals presented with more severe disease (mean score difference = 0.25) and had a higher risk of a poor treatment outcome compared to those who had not been incarcerated (risk ratio [RR] = 2.17). Among 138 with known incarceration dates, nearly three-quarters (73%) were diagnosed within two years of release, suggesting that infections were acquired while in prison. Among 7,101 household contacts aged 15–60 years, 121 (1.7%) had a history of incarceration and these had a higher prevalence of TB infection (prevalence risk ratio [PRR] = 1.33). The prevalence risk was similarly elevated in the subset who were incarcerated for only ≤ 3 months (PRR = 1.37). Incarceration leaves a lasting imprint on TB dynamics, driving more severe disease, poorer outcomes, and elevated household infection risk after release. Prisons act as reservoirs that amplify TB epidemics, underscoring the urgent need for control strategies that bridge prison and community health systems.

## Introduction

Prisons are high-risk environments for TB transmission due to overcrowding, poor ventilation, and inadequate healthcare. Prolonged close contact among persons deprived of liberty (PDLs) facilitates TB spread, while high rates of HIV infection, malnutrition, and substance use increase individual susceptibility.(1–5) Limited access to accurate diagnostic tools, inconsistent treatment protocols, and inadequate infection control measures further contribute to the spread of drug-resistant TB (DR-TB) and multidrug-resistant TB (MDR-TB).(2,6) Globally, PDLs face a more than tenfold higher risk of TB compared to the general population within the same communities. (2,7) The greatest disparity in TB incidence between PDLs and the general population is in Latin America, where the rapid expansion of the incarcerated population has driven an increase in national TB notifications.(8) In Peru, TB incidence rates among PDLs exceed those of the general population by more than 25-fold.(1,9)

While incarceration is a well-established risk factor for *Mycobacterium tuberculosis* (*Mtb*) infection and TB disease,(10) its full impact on community transmission is likely underestimated.(3) High turnover in prisons, coupled with TB’s long and variable latency period, means that many individuals may acquire infection while incarcerated but develop active disease after release. Cross-sectional studies identify TB genomic clusters that include individuals with and without a history of incarceration, indicating spillover from prisons into the community.(3,11,12) However, the extent of this transmission remains unclear. While the elevated TB risk among PDLs is well documented, research on those who develop disease after release from prison is limited—despite their key role in understanding how incarceration contributes to broader TB transmission dynamics.

In this study, we leveraged a prospective cohort of TB patients and their household contacts in Lima, Peru, to examine the relationship between incarceration history and TB outcomes after release. By integrating detailed epidemiological data and incarceration history, we aimed to provide insights into the interaction of TB transmission between prisons and the community.

## Results

### Index Patients

#### Incarceration history and established risk factors for TB transmission

Among 3,666 index patients aged 15–60, 188 (5.1%) reported a history of incarceration. Index patients with a history of incarceration were more likely to be male, smoke, drink alcohol, and have a positive sputum smear at the time of diagnosis (Table 1).

#### TB severity scores and treatment outcomes

Among 3,666 index patients aged 15–60, 513 (14%) had an unsuccessful treatment outcome. The mean TB severity score was 2.7 (standard deviation = 1.38). In the univariate analysis, those with a history of incarceration had higher TB severity scores at enrollment (mean difference [MD] = 0.30; 95% CI: 0.10–0.50) and experienced more unsuccessful treatment outcomes (risk ratio [RR] = 2.59; 95% CI: 1.99–3.38) compared to patients who had never been incarcerated. This association persisted after adjusting for age and sex (MD for severity score = 0.25; 95% CI: 0.05–0.47; RR for unsuccessful treatment outcomes = 2.17; 95% CI:1.63–2.90). Further adjustment for the DST profile of index patients had little impact on these associations (MD for severity score = 0.26; 95% CI: 0.06–0.47; RR for unsuccessful treatment outcomes = 2.10; 95% CI:1.59–2.76).

#### Time interval between release and TB diagnosis

**Figure 1:**
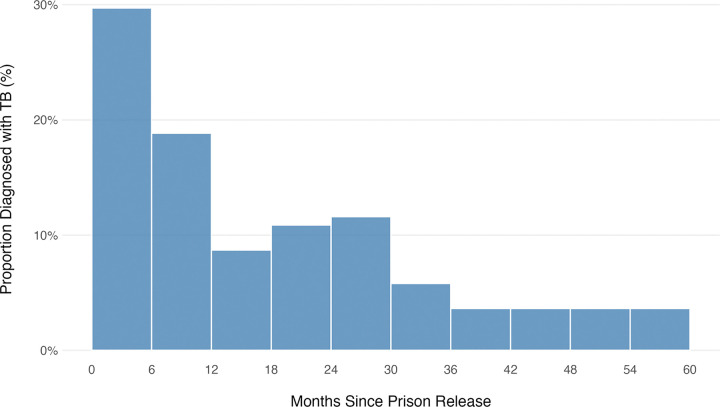
Frequency distribution of the time interval between release and TB diagnosis among TB index patients with a recent history of incarceration.

Among 188 index patients with a history of incarceration within 60 months prior to their TB diagnosis, 138 (73%) reported their time of release. Of these, 100 (73%) were released ≤ 24 months before their TB diagnosis. TB diagnoses did not occur uniformly over time (p < 0.001) but instead clustered around the time of release (Fig. 1). This pattern remained when we restricted the analysis to the 45 patients with DR-TB, of whom 35 (78%) were diagnosed with TB ≤ 24 months post-release. In a sensitivity analysis restricted to 72 index patients who had been incarcerated for 12 months or less, 51 (71%) were diagnosed with TB within 24 months of their release.

### Household contacts

Among 7,101 HHCs aged 15–60, 121 (1.7%) reported recent incarceration. In the univariate analysis, HHCs with a history of incarceration had a 1.34-fold higher prevalence risk of TB infection (95% CI: 1.20–1.49) at enrollment compared to those without such a history. This association remained nearly unchanged after adjusting for potential confounders (Prevalence Risk Ratio [PRR] = 1.33; 95% CI: 1.16–1.52).

**Figure 2:**
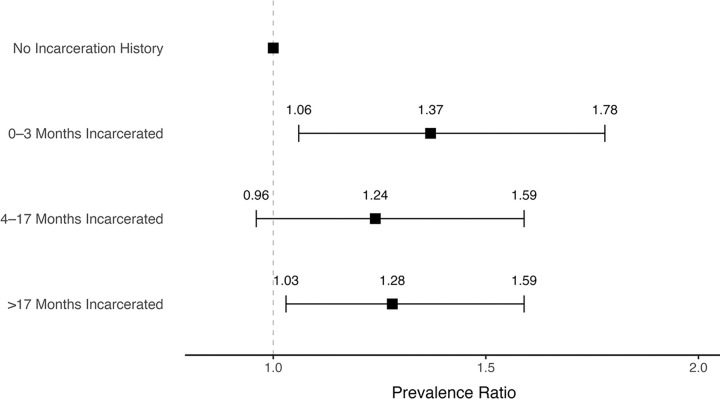
Associations between duration of incarceration and the baseline prevalence of TB infection among household contacts.

We did not observe a dose-response relationship between the duration of incarceration and TB infection risk. However, the risk of TB infection at enrollment was higher among those reporting any duration of incarceration (≤ 3 months vs. no history: PRR: 1.37; 95% CI: 1.06–1.78; 4–17 months vs. no history: PRR: 1.24; 95% CI: 0.96–1.59; >17 months vs. no history: PRR: 1.28; 95% CI: 1.03–1.59) (Fig. 2).

## Discussion

Compared to those without a recent incarceration history, index TB patients with such a history were more likely to have well-established risk factors for transmission, present with advanced disease at diagnosis, and experience worse treatment outcomes. Notably, over 70% of index TB patients with a recent incarceration history were diagnosed within two years of release. Household contacts with a recent incarceration history were also more likely to be infected at enrollment, even after adjusting for potential risk factors for TB infection. This increased risk persisted even among HHCs who had served only short sentences.

Our finding that a substantial proportion of TB patients with a recent incarceration history were diagnosed shortly after release aligns with the results of studies in Brazil, Paraguay, and France. Mabud et al. found that TB incidence among former PDLs in the Brazilian state of Mato Grosso do Sul between 2007 and 2013 was five times higher than baseline community incidence in the first year after release, declining over time.(11) Sequera et al. reported that among individuals incarcerated in Paraguay between 2010 and 2021, more than 40% of all TB cases were diagnosed after prison release. Within their cohort of former PDLs, TB notifications were highest in the first year following release and the elevated risk persisted for at least eight years. (3) Similarly, Niaux et al. reported that among 40 individuals who developed TB post-release in France from 2008 to 2020, 35% were diagnosed within two years after release.(18) This pattern of diagnosis suggests that many of these individuals acquired their TB infections while incarcerated.

Additional evidence for the spillover of TB from prisons comes from genetic studies. Research from Brazil and Thailand has demonstrated that TB patients with a history of incarceration are more likely to belong to large *Mtb* genetic clusters. Miyahara et al. reported that formerly incarcerated TB patients in Chiang Rai, Thailand were 4.5 times more likely than never-incarcerated individuals to be included in large genetic clusters and that 28% of individuals in these clusters had an incarceration history.(19) Similarly, Walter et al. found in Central West Brazil that 71% of genomic clusters involving never-incarcerated individuals also included people with a recent incarceration history. Moreover, over half (51%) of TB cases in non-incarcerated individuals were genetically clustered with those with incarceration history.(8) Using the *Mtb* WGS data of the index patients of our study, we previously showed that index patients with a history of incarceration were approximately 3 to 11 times more likely than others to transmit TB to another patient in our community-based cohort.(20) These findings support the hypothesis that prisons serve as reservoirs for TB and that former PDLs may be drivers of transmission.

Despite strong evidence linking incarceration to TB infection, we are not aware of studies that have specifically examined its prevalence among former PDLs. However, many studies that have conducted large-scale TB infection screenings in prisons have consistently reporting higher TB infection prevalence than in the general population, with increases varying widely (ranging from 5- to 83-fold) across countries.(1,7)

Several studies in Ethiopia and Brazil have assessed the association between incarceration duration and TB infection prevalence among PDLs, finding a stronger trend in Ethiopia than in Brazil. Chekesa et al. reported that in prisons in the East Wollega Zone, western Ethiopia, the prevalence of TB infection among PDLs incarcerated for more than 12 months was 16% higher than among those incarcerated for 12 months or less (61% vs. 45%).(21) Similarly, de Navarro et al. found that TB infection prevalence in Minas Gerais, Brazil was 3% higher among those incarcerated for at least 15 months compared to those with shorter stays (27% vs. 24%).(22) Notably, the reported 45% prevalence among PDLs with shorter prison stays in Ethiopia was considerably higher than the estimated TB infection prevalence in the general population (31%).(23) In our study, we also observed that HHCs with short prison stays already faced an increased risk of TB infection. These findings indicate that the extremely high TB burden in prisons contributes to substantial transmission risk, even for individuals incarcerated for short durations.

Some countries, including Brazil and Peru, have implemented active case-finding (ACF) programs incorporating TB symptom screening and chest radiography (CXR) to reduce TB transmission within prisons and prevent spillover into the community.(24–26) However, the timing of diagnosis in our cohort and in similar studies indicates that many TB-infected PDLs only develop active disease after release. This is especially likely for PDLs incarcerated for a short period, who make up a substantial proportion of the prison population in Latin America. For example, in a prison in Lima, housing approximately 8,500 PDLs, more than 50% were incarcerated for less than one year.(27) This included many individuals held in pre-trial detention, who may be imprisoned for months without a conviction due to overburdened court systems.(28,29) Thus, for a substantial proportion of PDLs, prisons may function as a revolving door of short detention, TB infection, and release, with the resulting transmission risk ultimately borne by the community. Consequently, an ACF program alone is unlikely to be sufficient to curb the TB spillover effect from prisons into the community.

Our study has several limitations. First, index TB patients enrolled in our prospective cohort study were likely to have better treatment outcomes than those in routine practice settings. Also, all HHCs were exposed to TB at home, so the prevalence of TB infection at enrollment among HHCs without a history of incarceration was higher than that in the general population of the same age group. Therefore, the effect sizes of the associations between individuals’ incarceration history and TB outcomes were likely underestimated compared to the true relative risks that would be observed in the general population. Second, recall bias regarding incarceration history may have introduced non-differential misclassification of exposure, which likely biased our results toward the null.

## Conclusion

Our study highlights the important role of incarceration in shaping TB transmission dynamics beyond prison walls, underscoring the need for interventions that bridge both prison and community settings. Implementing TB infection screening at prison entry or prior to release—paired with appropriate interventions such as TPT—may help mitigate spillover and reduce transmission in high-burden communities.

## Methods

### Study Setting and Data Collection

We conducted a prospective cohort study of TB patients and their household contacts in Lima, Peru. We identified individuals over 15 years of age with newly diagnosed pulmonary tuberculosis and invited them to participate as index patients. We confirmed TB diagnoses by sputum smear microscopy or mycobacterial culture. At enrollment, we collected detailed information on index patients’ sociodemographic characteristics, history of previous tuberculosis, tobacco and alcohol use, incarceration history, duration of symptoms before diagnosis, presence of cavitary lesions, and comorbidities including diabetes mellitus and HIV infection. The detailed study design and data collection process have been previously described. (13) Informed consent was obtained from all participants, and this study protocol was approved by the Harvard University Institutional Review Board and by the Research Ethics Committee of the National Institute of Health of Peru.

Household contacts (HHCs) of consenting index patients were enrolled within two weeks of the index patient’s recruitment. We referred those with signs or symptoms suggestive of TB disease to their local health center for further evaluation, including chest radiography and sputum smear testing. At enrollment, HHCs without a prior positive tuberculin skin test (TST) or history of tuberculosis disease had a TST to determine TB infection status. Those with an induration size ≥ 10 mm or a known positive TST were classified as TB-infected at enrollment. We also collected sociodemographic data from HHCs, including age, sex, incarceration history, comorbidities (e.g., diabetes mellitus and HIV status), Bacillus Calmette-Guérin (BCG) vaccination status, and use of isoniazid preventive therapy.

### TB severity score and TB treatment outcomes of index patients

We assessed TB severity in index patients using a modified Bantim TB Severity Score, incorporating the presence of cough, hemoptysis, night sweats, dyspnea, fever (> 37°C), and BMI (< 18 kg/m^2^).(14) Each of these factors contributed one point to the score, with an additional point assigned for BMI < 16 kg/m^2^, resulting in a maximum total score of seven. We followed index patients until they achieved TB cure, were lost to follow-up, died, or reached a maximum of five years, whichever occurred first. We classified treatment outcomes following World Health Organization (WHO) guidelines which define TB treatment outcomes as either successful (cure or treatment completion) or unsuccessful (treatment failure, loss to follow-up, or death).(15)

### Incarceration History

We asked index TB patients and HHCs whether they had been incarcerated for more than one day during the 60 months preceding enrollment. For those who reported incarceration, we collected the start and end dates (month and year) and calculated the time from release to disease diagnosis for index patients and the duration of incarceration of HHCs.

### Statistical Analysis

#### Index patients

We excluded index patients older than 60 years at enrollment from the analyses, as none of these patients reported a history of incarceration. We considered that TB patients with known risk factors for transmission may be more likely to contribute to community-level TB transmission. Therefore, we evaluated the association between incarceration history and well-established TB transmission risk factors using Fisher’s exact test. These factors included sex, tobacco use, alcohol use, sputum smear status at enrollment, and the presence of cavitary disease.(13)

We used linear and modified Poisson regression to assess associations between the incarceration history of index TB patients and (1) TB severity scores at enrollment and (2) treatment outcomes. We built univariate analyses and multivariate models adjusted for age and sex. Because previous studies have reported a high prevalence of drug-resistant TB (DR-TB) in prisons,(2) we performed a sensitivity analysis that adjusted for the drug susceptibility testing (DST) profiles of the index TB patients. We did not disaggregate these results by sex due to the very small number of female participants reporting an incarceration history.

We used the time interval from the patient’s reported date of release from prison until their disease diagnosis to evaluate whether TB disease in patients with an incarceration history was linked to infections that were acquired in prison. Since TB progression risk is highest in the first 24 months after infection,(16) we expected that if incarceration was driving TB risk, disease onset times in this group would be clustered near the time of prison release and incident disease would be less common as time since release increased. If incarceration had no impact, the time interval between release and disease onset would follow a uniform distribution. We tested this hypothesis using the Kolmogorov-Smirnov (K-S) test.(17) We also considered that some patients incarcerated for extended periods may have been infected early in their prison stay and therefore have a relatively constant risk of TB progression by the time they are released. To account for this, we conducted a sensitivity analysis restricting the sample to individuals incarcerated for 12 months or less.

### Household contacts

We excluded HHCs younger than 15 years or older than 60 years at enrollment from the analyses, as very few reported a history of incarceration. We used modified Poisson regression to evaluate the association between HHC incarceration history and their TB infection risk at enrollment. We constructed a univariate and multivariate model adjusting for index-patient and HHC characteristics identified a priori as potential modifiers of TB infection risk at enrollment. These included the index patient’s age, sex, smoking and drinking status, sputum smear grade, duration of cough symptoms, and presence of cavitary disease, as well as the HHC’s age, sex, BCG vaccination history, and socioeconomic status.

We examined the impact of the duration of incarceration on TB infection risk among HHCs at enrollment, categorizing the duration of incarceration into tertiles: ≤3 months, 4–17 months, and > 17 months.

## Figures and Tables

**Table 1:** Characteristics of index TB patients aged 15–60, stratified by recent incarceration history.

	Total		Without incarceration history		With incarceration history		P-values
Sex (n = 3,658)							< 0.001
Female	1,396	38%	1,392	40%	4	2%	
Male	2,262	62%	2,078	60%	184	98%	
Smoking status (n = 3,583)							< 0.001
Non-smoker	3,485	97%	3,327	98%	158	89%	
Smoker	98	3%	78	2%	20	11%	
Drinking status (n = 3,495)							< 0.001
Non-drinker	1,913	55%	1,877	57%	36	21%	
Drinker	1,582	45%	1,443	44%	139	79%	
Sputum smear status (n = 3,658)							0.045
Negative	1,011	28%	971	28%	40	21%	
Positive	2,647	72%	2,499	72%	148	79%	
Cavitary disease (n = 3,590)							0.276
No	2,605	73%	2,475	73%	130	69%	
Yes	985	27%	927	27%	58	31%	
Drug resistant profiles (n = 3,044)							0.503
Sensitive	1,875	62%	1,766	62%	109	63%	
Mono-resistance	487	16%	466	16%	21	12%	
Poly-resistance	235	8%	222	8%	13	8%	
Multidrug-resistance	447	15%	418	15%	29	17%	

## Data Availability

Reasonable data requests can be made to Megan Murray (megan_murray@hms.harvard.edu).
